# Effects of Mesenchymal Stem Cell Treatment on the Expression of Matrix Metalloproteinases and Angiogenesis during Ischemic Stroke Recovery

**DOI:** 10.1371/journal.pone.0144218

**Published:** 2015-12-04

**Authors:** Hyo Suk Nam, Il Kwon, Bo Hyung Lee, Haejin Kim, Jayoung Kim, Sunho An, Ok-Hee Lee, Phil Hyu Lee, Hyun Ok Kim, Hyun Namgoong, Young Dae Kim, Ji Hoe Heo

**Affiliations:** 1 Department of Neurology, Severance Hospital, Yonsei University College of Medicine, Seoul, Korea; 2 Severance Integrative Research Institute for Cerebral & Cardiovascular Disease, Yonsei University Health System, Seoul, Korea; 3 Department of Laboratory Medicine, Severance Hospital, Yonsei Cell Therapy Center, Yonsei University College of Medicine, Seoul, Korea; 4 Microbiology, Immunology and Molecular Genetics, University of California Los Angeles, Los Angeles, California, United States of America; National University of Singapore, SINGAPORE

## Abstract

**Background:**

The efficacy of mesenchymal stem cell (MSC) transplantation in ischemic stroke might depend on the timing of administration. We investigated the optimal time point of MSC transplantation. After MSC treatment, we also investigated the expression of matrix metalloproteinases (MMPs), which play a role in vascular and tissue remodeling.

**Methods:**

Human bone marrow-derived MSCs (2 × 10^6^, passage 5) were administrated intravenously after permanent middle cerebral artery occlusion (MCAO) was induced in male Sprague-Dawley rats. First, we determined the time point of MSC transplantation that led to maximal neurological recovery at 1 h, 1 day, and 3 days after MCAO. Next, we measured activity of MMP-2 and MMP-9, neurological recovery, infarction volume, and vascular density after transplanting MSCs at the time that led to maximal neurological recovery.

**Results:**

Among the MSC-transplanted rats, those of the MSC 1-hour group showed maximal recovery in the rotarod test (*P* = 0.023) and the Longa score (*P* = 0.018). MMP-2 activity at 1 day after MCAO in the MSC 1-hour group was significantly higher than that in the control group (*P* = 0.002), but MMP-9 activity was not distinct. The MSC 1-hour group also showed smaller infarction volume and higher vascular density than did the control group.

**Conclusions:**

In a permanent model of rodent MCAO, very early transplantation of human MSCs (1 h after MCAO) produced greater neurological recovery and decreased infraction volume. The elevation of MMP-2 activity and the increase in vascular density after MSC treatment suggest that MSCs might help promote angiogenesis and lead to neurological improvement during the recovery phase after ischemic stroke.

## Introduction

Mesenchymal stem cell (MSC) therapy might improve the functional outcome in stroke, and the efficacy of MSC therapy might differ depending on the timing of administration [1, 2]. Cell therapy is likely to be ineffective if it is administrated after an optimal time point. The Stem Cell Therapy as an Emerging Paradigm for Stroke guidelines encourage using a well-defined therapeutic window in stem cell research [3]. Although early rather than late MSC treatment might produce a greater effect on stroke recovery, only a few studies have investigated MSC transplantation during the hyperacute period [4–6]. A study conducted to compare effectiveness of intravenous (IV) MSCs treatment at the wide range of time windows from 3h to 72 h. The earliest transplantation showed smallest lesion volume [7]. Whereas, intra-arterial (IA) administration of a low dose of MSCs at 1 h after transient MCAO for 90 min did not reduce neurological deficits and infarction [8]. Therefore, optimal timing of MSCs is uncertain.

The mechanism by which MSC therapy benefits stroke recovery remains unclear. MSC therapy might promote stroke recovery through multiple mechanisms, including induction of angiogenesis and neurogenesis, prevention of apoptosis, and immunomodulation [9]. MSCs can stimulate angiogenesis and increase the total surface area of vessels in the ischemic boundary zone [10, 11]. MSCs secrete several cytokines and growth factors, including the pro-angiogenic molecules vascular endothelial growth factor (VEGF)/VEGF receptor 2 and angiopoietin/Tie2, directly or through endogenously induced mechanisms [11]. MSCs also secrete several matrix metalloproteinases (MMPs) [12, 13].

MMPs are a family of proteinases that degrade the extracellular matrix (ECM) [14, 15]. MMPs function as key enzymes under physiologic and pathologic conditions that require the degradation of ECM molecules, such as during cell differentiation, cell migration, and tissue injury and remodeling. MMPs have been suggested to play a dual role in stroke [16, 17]. Aberrantly secreted or activated MMPs exert harmful effects in ischemic injury by destroying the blood-brain barrier and compromising the integrity of the neurovascular unit [18, 19]. Conversely, MMPs might play beneficial roles during the recovery phase after cerebral ischemia by digesting degraded debris and mediating plasticity and remodeling [20]. MMP-2 is associated with angiogenesis, which might contribute to recovery after stroke [21]. Given the role played by MMPs in modulating the ECM and angiogenesis, the pro-angiogenic effect of MSC therapy could be mediated by MMPs.

In this context, we explored the optimal time point of MSC administration in cerebral ischemia and investigated the change in MMP activity after MSC treatment that can contribute to angiogenesis and recovery from ischemic stroke.

## Methods

### Ethics statement

This study was approved by the Severance Hospital Institutional Review Board (4-2008-0643) and written informed consents were obtained from all volunteers who agreed to the use of their cells for research purposes. Care and use of animals in our experiments were based on the institutionally approved protocol, in accordance with the Guide for the Care and Use of Laboratory Animals of the National Institutes of Health. The protocol for all animal experiments was approved by the Institutional Animal Care and Use Committee of Yonsei University College of Medicine (08–224). All surgeries and injections were performed under isoflurane inhalation anesthesia, and all efforts were made to minimize suffering. Animals were weighted daily and cirteria of humane endpoints were loss of their body weight more than 20%, any moribund or comatose animals with labored respiration, and end of experiments. Animals with a humane endpoint were sacrificed with intraperitoneal injection of overdose of Zoletil 50 (10 mg/kg, Vibac Laboratories, Carros, France).

### Induction of the middle cerebral artery occlusion

Male Sprague-Dawley rats weighing 270–400 g (minimum 276 g, maximum 364g) with the mean age of 9 to 11 weeks (mean 10 weeks) were subjected to permanent MCAO, as described previously [22]. Briefly, rats were anesthetized through inhalation of 1.5%–2% isoflurane mixed with 70% N_2_O and 30% O_2_. The body temperature was monitored using a thermometer and was maintained at 37 ± 0.5°C by using a homeothermic blanket (Harvard Apparatus Ltd., Watford, UK) during the surgery. Physiologic parameters such as pH, PaCO_2_, PaO_2_, and hemoglobin were assessed 10 min before and after MCAO. The left carotid artery of the rats was exposed through midline incision of the neck. After ligating the common carotid, the external carotid, and the pterygopalatine arteries, a 4–0 monofilament nylon suture (Ethicon, Edinburg, UK), which was rounded using a flame and treated with poly-L-lysine, was inserted into the internal carotid artery through a stump in the occluded external carotid artery. The middle cerebral artery was occluded permanently by advancing the nylon suture further up to 17.5 mm from the common carotid bifurcation. All animals were continuously monitored from surgery to recovery. After awakening from inhalation anesthesia, we observed for 1 hour. Daily observation and weight measurement were done during experiment.

### Neurologic assessment

Neurologic deficits were assessed using the rotarod test, the Longa score, and the modified Neurologic Severity Score (NSS) test before and after MCAO.

#### Rotarod test

The rats were placed on the rotarod cylinder, and the time for which the animals remained atop the rotarod was measured. The rotarod speed was slowly increased from 4 to 40 rpm over 5 min. A trial ended if the animal fell off the rungs or if it gripped the device and spun around for 2 consecutive revolutions without attempting to walk on the rungs. The animals were trained daily for 3 days before MCAO. The mean duration (in seconds) for which the animals remained on the rotarod cylinder was recorded based on the measurements of 3 trials one day before the surgery [23]. Follow-up tests`were performed at 1, 4, and 7 days (model 47700, Ugo Basile, Italy for Experiment 1) and 1, 4, 7, 10, and 14 days (Letica LE8500, Bioseb, France for Experiment 2) after MCAO.

#### Longa score

Rats were graded on a 5-point scale, which was modified from the Longa score [24]. Four items were evaluated and 1 point was assigned to each item when an animal (1) failed to grasp the edge of a table when it was hung by its tail; (2) failed to extend the right forepaw fully; (3) exhibited a circling motion toward the paretic side when attempting to walk; and (4) fell to the lateral side when pushed gently. The 4 items were summed to obtain a total score. Thus, an animal without any neurologic deficit received a score of 0, whereas an animal that exhibited maximal deficits received 4 points [24, 25].

#### Modified Neurologic Severity Score

The modified NSS test was performed before MCAO and 1, 4, 7, 10, and 14 days after MCAO. A modified NSS test is used to assess motor (6 points), sensory (2 points), balance (6 points), and reflex (4 points) functions. A rat without neurologic deficits receives a score of 0, whereas a rat that exhibits the most severe neurologic deficits receives 18 points [26].

### Study design

**Fig 1:**
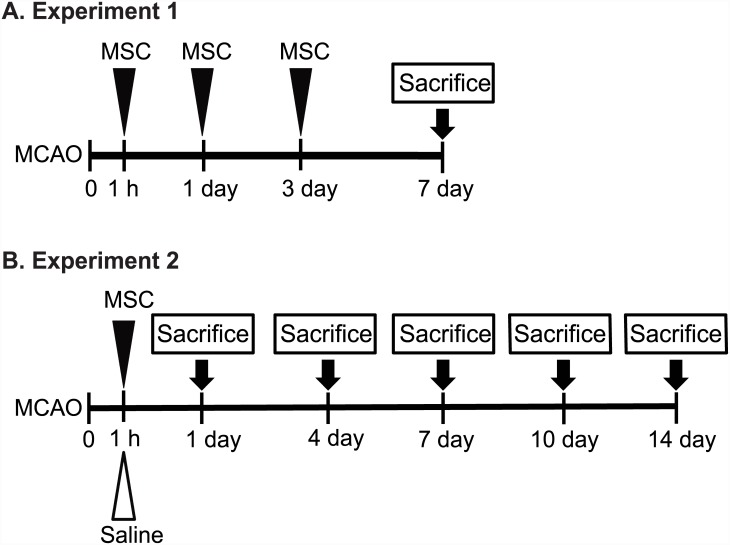
Study Design in Experiments 1 and 2. Experiment 1 was conducted to identify the time point of human mesenchymal stem cell (MSC) transplantation that led to maximal neurological recovery in rats after middle cerebral artery occlusion (MCAO). In Experiment 2, MSCs were administered at 1 h after MCAO, the time point identified in Experiment 1 as being optimal for enabling maximal neurological recovery. Infarction volume, neurological recovery, MMP-2 and MMP-9 activity, and vascular density after MCAO were compared between the MSC 1-hour group and the control group.

Experiment 1: We first determined the optimal time point of MSC administration after MCAO in rats. The optimal time point was defined as the time point of MSC injection that led to maximal neurologic recovery. In this experiment, MSCs were injected intravenously at 1 h (MSC 1-hour group, *n* = 7), 1 day (MSC 1-day group, *n* = 7), or 3 days (MSC 3-day group, *n* = 7) after MCAO. The control group received normal saline after MCAO (*n* = 8). Neurologic deficits were assessed using the rotarod test and the Longa score before MCAO and at 1, 4, and 7 days after MCAO. The MMP activity was measured after sacrificing the animals at 7 days after MCAO (Fig 1A).

Experiment 2: The results of Experiment 1 showed that maximal neurologic recovery occurred in the group that received MSC transplantation at 1 h after MCAO. Therefore, in Experiment 2, MSCs were administered at 1 h after MCAO. The rats were divided into a group that received an MSC injection at 1 h after MCAO (*n* = 34) and a control group that received an injection of normal saline (*n* = 34). We compared the neurologic deficits of the MSC-treated group and the control group after MCAO based on the results of the rotarod test and the modified NSS, infarction size, MMP-2 and MMP-9 activities, and vascular density (Fig 1B).

### Isolation and maintenance of human MSCs

Bone marrow aspirates were obtained from healthy volunteers, and mononuclear cells were isolated by means of Ficoll density centrifugation and then placed in 10-cm-diameter dishes. These cells were cultivated in low-glucose Dulbecco’s modified Eagle’s medium (DMEM, Gibco-BRL, Grand Island, NY, USA) containing 10% fetal bovine serum and 1% penicillin/streptomycin. The cells were maintained in a humidified incubator at 37°C in 5% CO_2_, and the medium containing non-adherent cells was replaced every 3 days. When the cultured cells reached 70%–80% confluence, they were trypsinized and subcultured. The human MSCs in passage 5 were used in our experiments [27]. We obtained written informed consents from all volunteers who agreed to the use of their cells for research purposes.

### Flow cytometry

Bone marrow-derived MSCs (1 × 10^6^) were trypsinized, washed with phosphate-buffered saline (PBS), and incubated with antibodies against CD14, CD29, CD31, CD34, CD44, CD45, CD73, CD90 (BD Biosciences, San Jose, CA, USA), CD105, and CD106 (Serotec, Cambridge, UK). Analysis was performed three separate cell preparations using a FACSCalibur flow cytometer (BD Biosciences).

### Transplantation of MSCs

Human bone marrow-derived MSCs (2 × 10^6^, passage 5) were suspended in 400 μL of saline. The suspended MSCs were slowly infused through the tail vein of rats by using a 1-mL syringe. The control rats received a slow 400-μL infusion of saline.

### Brain tissue preparation

All animals were sacrificed through transcardiac perfusion of cold heparinized normal saline by using a peristaltic pump when they were under deep anesthesia induced by intraperitoneal injection of Zoletil 50. The collected brains were sectioned into 2-mm-thick coronal blocks by using a rat brain matrix. The coronal blocks were stained with a 2% 2,3,5-triphenyltetrazolium chloride (TTC) solution, and infarction sizes were measured in the 1^st^, 3^rd^, 5^th^, and 7^th^ blocks. The 4^th^ block was cut into the left and right hemispheres, embedded in Tissue-Tek OCT compound (Miles, Inc., Elkhart, IN, USA), quickly frozen using 2-methylbutane and dry ice, and stored at –80°C until analysis.

### TTC staining and infarction-volume measurement

Fresh blocks were stained with the TTC solution in order to determine infarct volumes. Briefly, each block from the frontal pole was incubated in a 2% TTC solution at 37°C for 30 min, fixed with 10% buffered formalin solution, and scanned using a 600-dpi flatbed scanner. The infarcted areas and whole brain areas in each block were measured by an investigator who was blinded to the animal group; the areas were measured using Medical Image Processing, Analysis, and Visualization (MIPAV) software (Computer and Information Technology, National Institutes of Health, Bethesda, MD, USA). The infarction volume was calculated by numerically integrating the values from individual blocks (1^st^, 3^rd^, 5^th^, and 7^th^ blocks) and dividing by the whole brain area [25].

### Gelatin zymography for measuring MMP activities

MMP-2 and MMP-9 activities were measured using gelatin zymography. The sections were placed in 120 μL of homogenizing buffer (1% Triton X-100, 50 mmol/L Tris-HCl [pH 7.5], 75 mmol/L NaCl, 1 mmol/L phenylmethyl sulfonyl fluoride (PMSF, Sigma-Aldrich, St. Louis, MO, USA) and were centrifuged at 9000 rpm for 20 min at 4°C. The supernatant was obtained from the centrifuged mixture and stored at −80°C. The protein concentration of stored samples was measured using the Bradford method (Bio-Rad Laboratories, Hercules, CA, USA) and a bovine gamma globulin standard. Zymography was performed according to previously published methods; we mixed 50 μg of purified protein extracts with an equal volume of sample buffer (pH 6.8, 80 mmol/L Tris-HCl, 4% sodium dodecyl sulfate [SDS], 10% glycerol, 0.01% bromophenol blue) [18, 22], and then measured the gelatinolytic activities of samples by using 8% SDS-polyacrylamide gels containing 1% gelatin. Sample gels were rinsed in 150 mL of 2.5% Triton X-100 (15 min × 3) and incubated at 37°C in 250 mL of 50 mmol/L Tris-HCl buffer (pH 7.5, 10 mmol/L CaCl_2_, 0.02% NaN_3_) for 66 h. After incubation, the gels were stained with a 0.1% amido-black solution containing acetic acid, methanol, and distilled water (volume ratio 1:3:6) for 1 h, and then destained by washing 4 times for 20 min with the same solution without amido black. The gels were scanned using a flatbed scanner and the gelatinolytic bands were quantified using Scion Image software (Scion Corporation, Frederick, MD, USA) and a gel-plotting macro. Measured values were standardized relative to the sample values of recombinant MMP-2 and MMP-9 in each gel.

### Immunostaining

Immunohistochemistry was performed for collagen type 4 (1:800; Developmental Studies Hybridoma Bank, Iowa City, IA, USA). Frozen sections were fixed with methanol for 10 min and immersed in 100 mmol/L of glycine in PBS for 10 min. To reduce nonspecific staining, the sections were washed with a PBS solution and incubated with Blotto (5% hydrated nonfat dry milk containing 1% BSA) for 20 min. After blocking, the sections were incubated with primary antibodies overnight at 4°C under humidified conditions. After washing with PBS, a biotinylated anti-mouse secondary antibody (Vector Laboratories, Burlingame, CA, USA) was applied for 30 min at 37°C. Avidin-biotin complexes were generated using streptavidin-horseradish peroxidase (Vector Laboratories), the peroxidase signal was developed using a 3,3ʹ-diaminobenzidine solution (Vector Laboratories), and the immunostained cells were analyzed using bright-field microscopy.

To perform immunofluorescence double-labeling, brain tissues were fixed and permeabilized and then stained with primary antibodies against MMP-2 (1:200; Millipore, Milford, MA, USA), NeuN (1:100; Chemicon, Temecula, CA, USA) for neurons, OX-42 (Serotec; Raleigh, NC, USA) for microglia, glial fibrillary acidic protein (GFAP, 1:200; Santa Cruz Biotechnology, Santa Cruz, CA, USA) for astrocytes, or collagen type 4 (1:800, Developmental Studies Hybridoma Bank) for microvessels. All tissues were then incubated with Cy3-labeled anti-rabbit IgG and either FITC-labeled anti-goat or anti-mouse IgG (all from Jackson Immunoresearch Laboratories, West Grove, PA, USA). Images were acquired using an Axio Imager.D2 (Zeiss, Oberkochen, Germany) equipped with a high-resolution camera (AxioCam HRC, Zeiss).

### Vascular density

We measured and compared vascular density at 10 days after MCAO between the MSC 1-hour group and the control group based on immunohistochemical staining for collagen type 4. Using the computer-assisted stereological toolbox system (CAST; Olympus Danmark A/S, Albertslund, Denmark), the number and diameter of the vessels were semi-automatically measured at 120–260 randomly sampled points per coronal section of the whole brain, which constituted a 10% fraction of the section. Vascular density is expressed as the number of vessels per unit area (mm^2^) [28].

To reveal the effect of MMP-2 in angiogenesis, we performed a randomized placebo-controlled, blind experiment. Permuted-block randomization with a block size of 4 was generated to compare vascular density between animals that were treated with MSCs and a selective MMP-2 inhibitor (sc-204092, Santa Cruz Biotechnology) and those with MSCs and placebo. Under inhalation anesthesia with 1.5%–2% isoflurane, animals’ head were placed and fixed on a stereotaxic frame, and bregma was exposed with a midline incision. Bone was drilled and removed at 1.5 mm lateral and 0.3 mm posterior to the bregma. A Hamilton needle (32 gauge) was inserted through the burr hole 4.5 mm deep below the dura to reach to the lateral ventricle [29]. The selective MMP-2 inhibitor or placebo was administered at 1 h after MCAO. The selective MMP-2 inhibitor 250 μg/kg or vehicle was intracerebroventricularly infused for 10 min [30]. During the intracerebroventricular infusion, 2 × 10^6^ of MSCs were injected through the tail vein according to the previous studies [29–31]. Vascular densities were measured at 10 days after MCAO. Gelatin zymography was performed to measure the changes of MMP-2 activity in rat brain obtained at 10 days. Investigators who were blinded to the treatment group assessed vascular density and gelatin zymography.

### Statistical analysis

SPSS for Windows (Version 18.0, SPSS Inc., Chicago, IL, USA) was used for statistical analysis. For continuous variables, the mean and standard deviation (SD) were calculated and independent-sample *t* tests were used for comparisons. Differences in the results of the neurologic exams among groups were determined using the repeated measurement analysis of variance (ANOVA) test followed by Tukey’s post-hoc analysis. *P* < 0.05 was considered statistically significant.

## Results

### MSC identity confirmation using flow cytometry

Flow-cytometric analysis of donor MSCs showed that the MSC-specific antigens CD105 and CD90 [32] were expressed in 99.48% and 99.94% of all cells, respectively. By contrast, the hematopoietic progenitor cell antigen CD34 was expressed in only 2.64% of the total cells (S1 Fig).

### Physiologic parameters

The physiologic parameters mean arterial blood pressure, pH, PaCO_2_, PaO_2_, and hemoglobin were not different between the MSC-treated and control groups before and after MCAO (S1 Table).

### Experiment 1

#### Mortality and neurologic outcome

**Fig 2:**
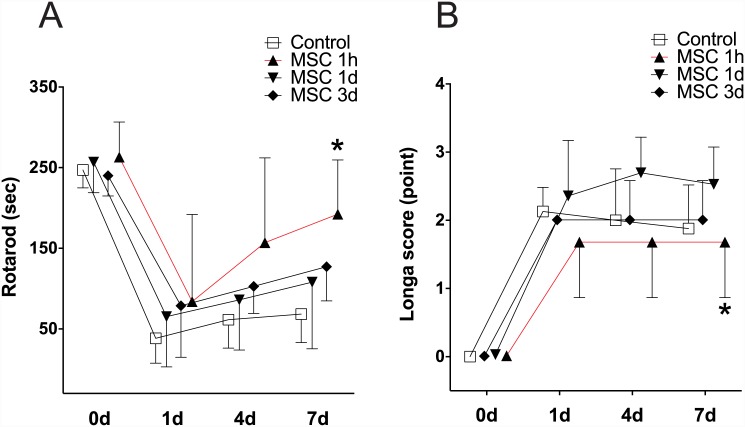
Behavioral Tests in Experiment 1. MSCs were injected intravenously at 1 h (MSC 1-hour group, *n* = 7), 1 day (MSC 1-day group, *n* = 7), or 3 days (MSC 3-day group, *n* = 7) after MCAO. The control group received normal saline after MCAO (*n* = 8). Behavioral tests were performed using the rotarod test (A) and by measuring the Longa scores (B) before MCAO and at 1, 4, and 7 days after MCAO. At 7 days after MCAO, the results of the rotarod test showed significant functional recovery in the rats treated with MSCs at 1 h. The Longa scores were also more favorable in the MSC 1-hour group than in the control group (*P < 0.05).

Of the 29 rats used, 2 (7.4%) died because of large hemispheric infarctions after MCAO (one of the MSC 1-hour group and one of the MSC 1-day group). Therefore, we performed neurologic assessments on the 27 surviving animals (6, MSC 1-hour group; 6, MSC 1-day group; 7, MSC 3-day group; 8, control group). Before MCAO, the mean duration of holding on the rotarod cylinder did not differ among the groups (264 ± 44 s in the MSC 1-hour group, 247 ± 38 s in the MSC 1-day group, 230 ± 25 s in the MSC 3-day group, and 247 ± 22 s in the control group, *P* = 0.453). By contrast, the mean durations of holding on the rotarod cylinder at 1, 4, and 7 days after MCAO were distinct among the groups (df = 3, F = 3.431, *P* = 0.034). A post-hoc analysis revealed that the MSC 1-hour group showed greater recovery in the rotarod test than did the control group (*P* = 0.023). The Longa score also differed among the groups (df = 3, F = 1.597, *P* = 0.03), and this score of the MSC 1-hour group was lower than that of the MSC 1-day group in the post-hoc analysis (*P* = 0.018) (Fig 2).

#### MMP activities

**Fig 3:**
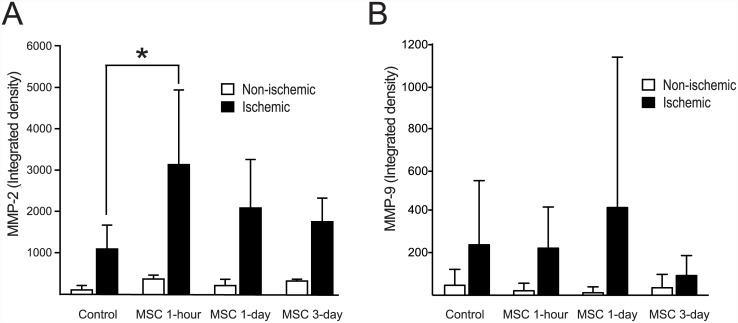
MMP-2 and -9 Activities Revealed Using Zymography. MMP activities in the ischemic hemisphere were measured using gelatin zymography. The MSC 1-hour group showed higher MMP-2 activity than did the control group (*P* = 0.028). However, MMP-2 activity was not distinct between the MSC 1-day or 3-day group and the control group (A). MMP-9 activity was not different between the MSC-treated groups and the control group (B).

MMP activities in the ischemic hemisphere were measured using gelatin zymography. The MMP-2 activity of the MSC 1-hour group was higher than that of the control group (3130 ± 1807 vs. 1087 ± 579, *P* = 0.028). The MMP-2 activities of the MSC 1-day and 3-day groups were not different from those of the control group. In contrast to MMP-2 activity, MMP-9 activity did not differ between the MSC-treated groups and the control group (Fig 3).

### Experiment 2

#### Mortality and neurologic outcomes

**Fig 4:**
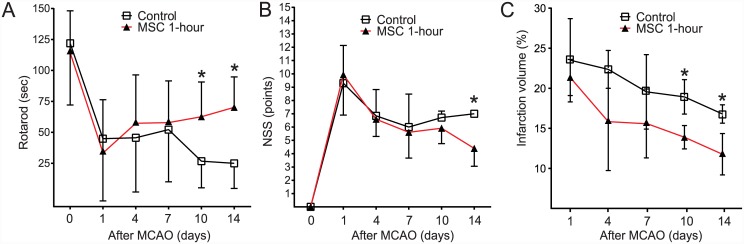
Neurological Recovery and Infarction Volume. The surviving animals (MSC 1-hour group, *n* = 32; control group, *n* = 27) were subjected to behavioral tests by using the rotarod test and by measuring the modified Neurologic Severity Score (NSS) at baseline and at 1, 4, 7, 10, and 14 days after MCAO. In the rotarod test, the MSC 1-hour group exhibited statistically significant functional recovery at 10 and 14 days (A). The modified NSS at 14 days also revealed greater recovery in the MCS 1-hour group than in the control group. Because the modified NSS scores at 14 days of all 4 rats in control group were 7, the error bar could not be drawn (B). The infarction volume was smaller in the MSC 1-hour group than in the control group at 10 and 14 days after MCAO (C).

Of the 68 rats used, 9 (13.2%) died because of large hemispheric infarction within 1 day after MCAO (2 of the MSC 1-hour group and 7 of the control group, *P* = 0.15). Baseline results of the rotarod test were not different between the groups (117 ± 32 s in the MSC 1-hour group, 122 ± 50 s in the control group, *P* = 0.552). However, the performance of the MSC 1-hour group on the rotarod cylinder was superior to that of the control group at 10 days after MCAO (63 ± 28 vs. 27 ± 21 s, *P* = 0.013) and also at 14 days after MCAO (70 ± 25 vs. 25 ± 20 s, *P* = 0.027) (Fig 4A). The modified NSS did not differ between the MSC 1-hour group and the control group until 10 days after MCAO; however, at 14 days, the recovery exhibited by the MSC 1-hour group was superior to that of the control group (4.4 points in the MSC 1-hour group vs. 7 points in the control group, *P* = 0.01) (Fig 4B).

#### Infarction volumes

Until 7 days after MCAO, infarction volumes did not differ between groups. However, at 10 and 14 days after MCAO, the infraction volume was smaller in the MSC 1-hour group than in the control group (10 days: 13.9% ± 1.5% vs. 18.9% ± 2.1%, *P* = 0.028; 14 days: 11.8% ± 2.58% vs. 16.8% ± 1.1%, *P* = 0.01) (Fig 4C).

#### MMP activities

**Fig 5:**
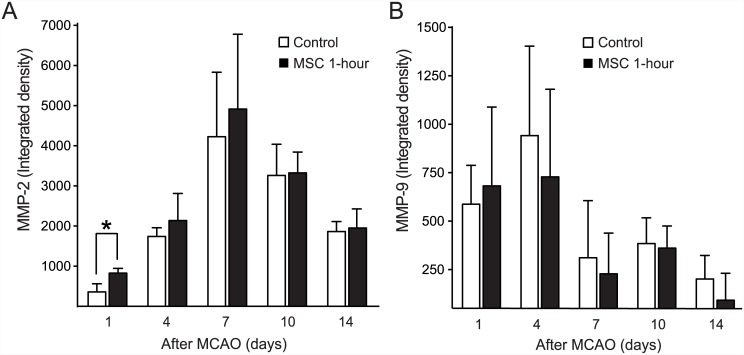
MMP-2 and -9 activities in the Ischemic Hemisphere. The MMP-2 activity in the MSC 1-hour group was significantly higher only at day 1 after IV infusion. Although remains higher subsequently, it did not remain statistically significant (A). By contrast, MMP-9 activity in the MSC 1-hour group was not significantly different from that in the control group. The increased MMP activities measured after MCAO decreased starting from 10 days (MMP-2) and 7 days (MMP-9) (B).

MMP-2 activities increased over time in both the MSC 1-hour group and the control group until 7 days after MCAO. Comparison of the MSC 1-hour and control groups showed that MMP-2 activity in the MSC 1-hour group was significantly higher than that in the control group at 1 day after MCAO (828 ± 119 vs. 360 ± 203, *P* = 0.002). By contrast, MMP-9 activities of the MSC 1-hour and control groups were not different. Increased MMP activities measured after MCAO were decreased starting from 10 days (MMP-2) and 7 days (MMP-9) (Fig 5).

#### Vascular density after MSCs treatment

**Fig 6:**
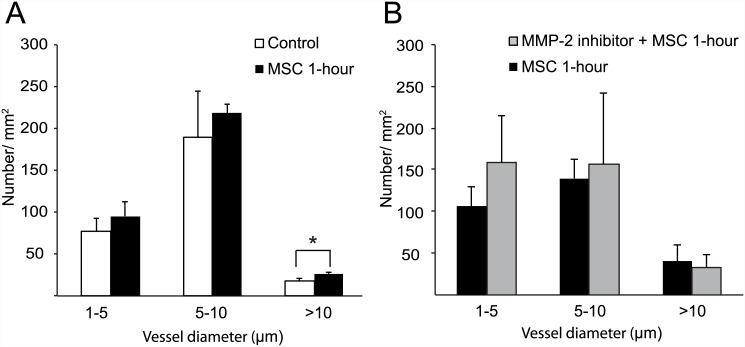
Vascular Density. Using the computer-assisted stereological toolbox system, the number and the diameter of vessels were measured based on immunohistochemical staining for collagen type 4 at 10 days after MCAO. The number of the larger capillaries (>10 μm) was significantly higher in the MSC 1-hour group than the control (P = 0.036) (A). After the intracerebrolventricular administration of a selective MMP-2 inhibitor or placebo, vascular density was not significantly different in all ranges of vessel diameter (B).

Vascular density, which was determined based on collagen type 4 staining, was increased to a greater extent in the MSC 1-hour group than in the control group at 10 days after MCAO. Quantification revealed that the number of large-sized vessels (>10 μm) was significantly higher in the MSC 1-hour group than in the control group (P = 0.036) (Fig 6A).

#### Vascular density after MMP-2 inhibition

During 10 days of follow-up after the intracerebrolventricular administration of a selective MMP-2 inhibitor or placebo, 6 of the10 MMP-2 inhibitor-treated rats and 7 of the 10 placebo-treated rats were died. Of survived rats, vascular density was not significantly different between groups [p = 0.261 for small-sized capillaries (1–5 μm), p = 0.793 for medium-sized capillaries (5–10 μm), and p = 0.667 for large-sized vessels (>10 μm)] (Fig 6B).

#### Immunofluorescence staining

**Fig 7:**
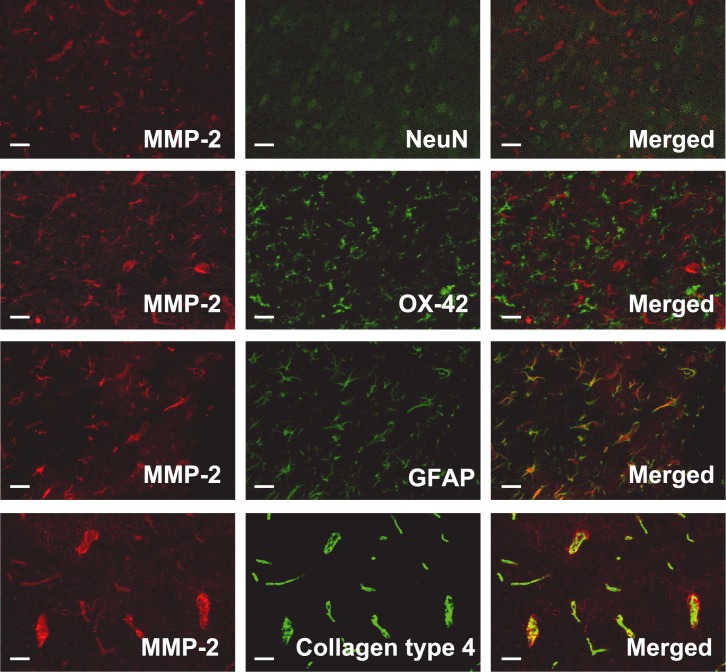
Immunofluorescence staining for MMP-2, NeuN, GFAP, OX-42, and Collagen type 4. Immunofluorescence staining showed that MMP-2 was not colocalized with neurons (NeuN) or microglia (OX-42), but it was colocalized with astrocytes (GFAP) and and was detected at the outer rim of the capillary basement membrane (collagen type 4), Scale bar = 50 μm.

Immunofluorescence staining revealed that MMP-2 was neither colocalized with NeuN nor OX-42, but was colocalized with GFAP and was detected at the outer rim of the staining for collagen type 4, which suggested that MMP-2 was expressed in astrocytes (Fig 7).

## Discussion

This study showed that maximal neurological improvement occurred when MSCs were transplanted very early (1 h) after MCAO, and that the improvement was apparent at about 10 days after treatment.

Therapeutic time window of IV MSC transplantation is unknown. Earlier treatment with MSCs mediates neuroprotection in acute period and may allow functional recovery. Only a few studies have investigated the effect of MSC transplantation during the hyperacute period (<3 h). Most previous studies tested the efficacy of IV MSCs therapy form 24 h to 1 month [23, 26, 33, 34]. Although, transplantation of MSCs at 24 h or delayed time injection showed neurological recovery, it did not decrease the infarction volume in a statistically significant manner [6, 35–38], which suggests that MSC transplantation at this time point is likely insufficient for reverting tissue damage.

In contrast to previous studies, the transplantation of MSCs at 1 and 3 days did not improve functional outcome in this study. This might be partly ascribed to the difference of a stroke model. We used a permanent MCAO model while others did a transient MCA model. The permanent MCA model induces more severe ischemic injury and less delivery of MSCs to infarct core than the transient MCAO model. We also used later passages of MSCs (5 passage MSCs). These factors might have resulted in less effect in rats treated at 1 or 3 days in our experiment. The different recipient species, cell sources of donors, and doses of MSCs might also influence the result (S2 Table).

IA delivery of MSCs at 1 h after 90 min of transient MCAO using a lower dose of MSCs (1X10^5^) was not superior to controls, while the IA delivery at 24 h after transient MCAO showed superior functional outcomes and smaller infarctions as compared to controls [8]. The number of cells (1X10^5^) in that study was chosen based on arterial blood flow measurement using a laser Doppler study, which did not show decrease in the cerebral blood flow. Although the cells that were delivered by IA route might not occlude the artery or arteriole, they could have impeded microcirculation at the capillary level. The ischemic penumbra area is much more sensitive to decrease in blood flow during hyperacute period than later stage of cerebral ischemia. Consequently, the beneficial effects of MSCs might be neutralized by the negative effect of impeded microcirculation during hyperacute period (1 h after transient MCAO), but not at 24 h after transient MCAO. This issue may be better resolved by the direct comparison of IV and IA delivery at the same time.

Our observations showing an increase of vascular density at 10 days after MCAO suggest that MSC treatment in the hyperacute stage might trigger angiogenesis, which, in turn, would result in recovery from ischemic injury and functional improvement. Previous study showed that IV injection of human MSCs (1 X 10^6^) at 24 h after MCAO can promote endogenous VEGF secretion, VEGF receptor 2 expression and increased angiogenesis [10]. Angiogenesis is correlated with functional improvement in both animal models of stroke and human stroke [39]. Stroke patients exhibiting a high cerebral blood vascular density showed better prognosis and longer survival than did patients exhibiting lower vascular density [40]. Cerebral ischemia itself induces neovascularization within the ischemic boundary zone [41], and angiogenesis is essential for repairing injured cerebral tissues.

Proliferation of endothelial cells begins as early as 12–24 h after a stroke, and the increase in the number of vessels is apparent 3 days after ischemic injury [42]. Further formation of new stable vasculature takes a few weeks (7–28 days) [43]. Newly formed vessels improve tissue perfusion and enhance subsequent angiogenesis and promote functional recovery after stroke [10]. Before endothelial proliferation begins, most of the well-known angiogenic factors including VEFG and angiopoietin-2 show increased expression as early as a few hours after ischemia [44]. Besides influencing recognized angiogenic factors, MSC transplantation triggers the secretion of unidentified factors and might thus enhance the angiogenic process [45].

One of the critical events required for successful angiogenesis is extracellular proteolysis, which involves the degradation of the vascular basement membrane and the activation of cytokines [46]. Therefore, we measured the activity of MMP-2 and MMP-9 after MSC transplantation. MMPs are key proteolytic enzymes that mediate the degradation of the vascular ECM. The expression levels of MMP-9 and MMP-2 have been reported to start increasing from 12 h to 2 days and from 2 to 5 days after cerebral ischemia, respectively [15]. These temporal profiles of expression after cerebral ischemia suggest a role of MMP-9 in secondary tissue damage and that of MMP-2 in tissue repair [14].

MMP-2 critically affects vascular development and angiogenesis. Angiogenesis is reduced in MMP-2 knockout mice and following treatment with natural or synthetic inhibitors of MMP-2 [21]. A clinical study showed that basal MMP-2 levels were higher in patients exhibiting stable or recovering symptoms than in other patients [47]. In this context, early MMP-2 elevation after ischemia might be beneficial because MMP-2 is a crucial protease involved in blood vessel remodeling through angiogenesis [10, 44, 48]. However, vascular density was not changed by intracerebroventricular infusion of a selective MMP-2 inhibitor in this study. This finding suggests that pro-angiogenic effects of MSCs transplantation may not be solely mediated by MMP-2. MSC behave as a small biochemical factory producing trophic factors (e.g., VEGF/Flk1 and Ang1/Tie2) or induce endogenous VEGF and VEGFR2 expression [44, 49]. These factors also might be related with promoting angiogenesis. Further experiments may be required to address the mechanism of increased vascular density after MSCs transplantation.

This study has limitations. First, producing the beneficial effects of early MSC transplantation in patients with acute ischemic stroke might not be practical, because expanding human MSCs in order to perform autologous transplantation takes one month. Thus, further studies are required to bridge the gap between animal experiments and clinical trials. Second, in this study, we used a rat model and permanent MCAO, and results could differ between species or between permanent and transient ischemic stroke models. Third, we used later passages of MSCs while the use of earlier passage of cell may show better efficacy. Culturing up to later passages can increase the number of cells. It had been reported that MSCs with passage 5 or 6 are the earliest passages to achieve a sufficient IV dose of MSCs in human [50]. We wanted to balance between the efficacy of MSCs and limited amounts of human MSCs. Finally, MSC is reported as relatively immune privileged in allogeneic transplantation and allogeneic transplantation of MSCs has been reported to be safe and effective. We used xenogeneic model using human MSCs. Recent meta-analysis revealed that the effect was better when the source of MSCs was human than it was rat or mouse even in xenogeneic approach [51]. Because allogeneic “off the shelf” MSCs can be used in clinical settings allowing for very early transplantation, IV infusion of allogeneic MSCs may be possible in clinical trials.

In conclusion, our study showed that very early transplantation of human MSCs in a permanent model of rodent MCAO improved neurologic outcomes and decreased infarction volume after ischemia. The increases in MMP-2 activity and vascular density after MSC transplantation suggest that MSC treatment may help promote angiogenesis, which would result in neurologic improvement during the recovery phase.

## Supporting Information

S1 Checklist ARRIVE Guidelines Checklist. (PDF)

S1 Fig Characteristics of MSCs. Flow-cytometric analysis for donor MSCs showed that the MSC-specific antigens CD105 and CD29 were expressed in 91.14% and 81.83% of all cells, respectively. By contrast, the hematopoietic progenitor cell antigen CD34 was expressed in only 0.27% of the total cells. Analysis was performed three separate cell preparations using a flow cytometer.
(PNG)

S1 Table Physiologic Parameters Between Groups. (DOCX)

S2 Table Previous Experiments of Intravenous MSC Injection Within 3 Days After Induction of the Middle Cerebral Artery Occlusion. (DOCX)
