# Correction: Effects of Mesenchymal Stem Cell Treatment on the Expression of Matrix Metalloproteinases and Angiogenesis during Ischemic Stroke Recovery

**DOI:** 10.1371/journal.pone.0146628

**Published:** 2016-01-04

**Authors:** Hyo Suk Nam, Il Kwon, Bo Hyung Lee, Haejin Kim, Jayoung Kim, Sunho An, Ok-Hee Lee, Phil Hyu Lee, Hyun Ok Kim, Hyun Namgoong, Young Dae Kim, Ji Hoe Heo

An affiliation for the fifth and sixth authors is missing. Jayoung Kim and Sunho An are both affiliated with: Brain Korea 21 Plus Project for Medical Science, Yonsei University, Seoul, Korea
