# Supplementary figures and images for: Effects of Mesenchymal Stem Cell Treatment on the Expression of Matrix Metalloproteinases and Angiogenesis during Ischemic Stroke Recovery
Source: PLoS One. 2015 Dec 4;10(12):e0144218. doi: 10.1371/journal.pone.0144218 (PMC4670145; doi:10.1371/journal.pone.0144218)

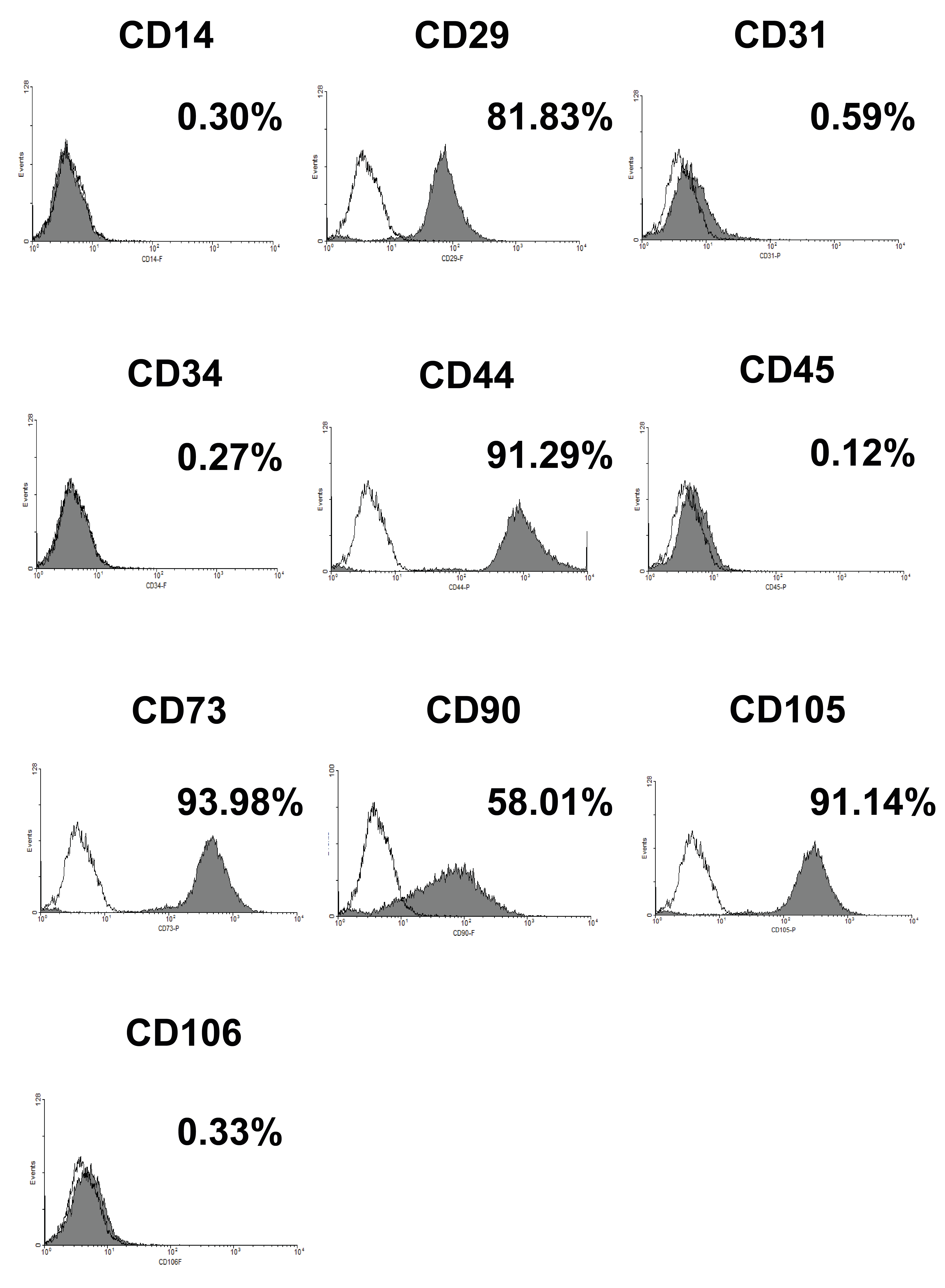

Supplement: S1 Fig — Flow-cytometric analysis for donor MSCs showed that the MSC-specific antigens CD105 and CD29 were expressed in 91.14% and 81.83% of all cells, respectively. By contrast, the hematopoietic progenitor cell antigen CD34 was expressed in only 0.27% of the total cells. Analysis was performed three separate cell preparations using a flow cytometer. (PNG) [file pone.0144218.s002.png]
