# Supplementary material for: Effects of Mesenchymal Stem Cell Treatment on the Expression of Matrix Metalloproteinases and Angiogenesis during Ischemic Stroke Recovery
Source: PLoS One. 2015 Dec 4;10(12):e0144218. doi: 10.1371/journal.pone.0144218 (PMC4670145; doi:10.1371/journal.pone.0144218)
Supplement: S1 Table — (DOCX) [file pone.0144218.s003.docx]

**Supplemental Table 1. Physiologic Parameters Between Groups**

|  |  | PH | PaO_2_ (mmHg) | PaCO_2_ (mmHg) | MAP (mmHg) | Hb (gm/dL) |
| --- | --- | --- | --- | --- | --- | --- |
| MSCs group (n = 5) |  |  |  |  |  |  |
|  | Before MCAO | 7.42 ± 0.05 | 137.80 ± 15.99 | 47.00 ± 9.27 | 96.33 ± 10.46 | 13.54 ± 1.96 |
|  | After MCAO | 7.37 ± 0.08 | 132.75 ± 12.61 | 49.08 ± 6.21 | 90.47 ± 23.90 | 14.05 ± 0.76 |
|  | After MSCs injection | 7.43 ± 0.06 | 132.40 ± 12.62 | 43.94 ± 9.30 | 87.80 ± 7.11 | 14.36 ± 0.51 |
| Control group (n = 5) |  |  |  |  |  |  |
|  | Before MCAO | 7.41 ± 0.04 | 140.17 ± 15.43 | 46.85 ± 8.30 | 98.76 ± 9.53 | 14.62 ± 0.82 |
|  | After MCAO | 7.37 ± 0.07 | 129.00 ± 13.77 | 48.92 ± 5.39 | 92.14 ± 19.72 | 14.24 ± 0.78 |
|  | After saline injection | 7.44 ± 0.05 | 136.00 ± 14.32 | 44.28 ± 8.37 | 82.86 ± 10.28 | 14.55 ± 0.65 |

MSCs, mesenchymal stem cells; MCAO, middle cerebral artery occlusion; MAP, mean arterial blood pressure; Hb, hemoglobin.
