# Supplementary material for: Effects of Mesenchymal Stem Cell Treatment on the Expression of Matrix Metalloproteinases and Angiogenesis during Ischemic Stroke Recovery
Source: PLoS One. 2015 Dec 4;10(12):e0144218. doi: 10.1371/journal.pone.0144218 (PMC4670145; doi:10.1371/journal.pone.0144218)
Supplement: S2 Table — (DOCX) [file pone.0144218.s004.docx]

**Supplemental Table 2. Previous Experiments Of Intravenous MSC Injection Within 3 Days After Induction Of The Middle Cerebral Artery Occlusion.**

| Injection time | Species | Model | MSC | Passage | Dose | Reduced infarction volume | Improved functional outcome | Ref |
| --- | --- | --- | --- | --- | --- | --- | --- | --- |
| 24h or 7 days | Wistar rat | 2 h transient MCAO | Allogenic  rat  MSC | NA | 3x10^6^ and 1x10^6^ | No | Yes in Adhesive removal test, rotarod test, and NSS | Stroke. 2001;32:1005-1011. |
| 24h | Wistar rat | 2 h transient MCAO | Human MSC | NA | 3X10^6^ | No | Yes in Adhesive removal test, and mNSS | Neurology. 2002;59:514-523. |
| 24h | Wistar rat | 2 h transient MCAO | Allogenic rat MSC | NA | 3X10^6^ | No | Yes in Adhesive removal test and rotarod test | J. Neurosci. Res. 2003;73:778-786. |
| 24h | Wistar rat | 2 h transient MCAO | Human MSC | 3 to 5 | 1x10^6^ | NA | NA, increased angiogenesis | Circ. Res. 2003;92:692-699. |
| 3h, 6h, 12h, 24h, and 72 h | SD rat | 45 min transient MCAO | Autologous rat MSC | NA | 1X10^7^ | Yes | Yes in water maze test and rotarod test | Brain Res. 2004;1007:1-9. |
| 6h | SD rat | 90 min transient MCAO | Noggin-BMSCs | 5 | 5X10^6^ | Yes | Yes in limb placement test and the treadmill stress test | J. Neurosci. Res. 2011;89:1194-1202. |
| 24h | Wistar rat | Permanent MCAO | Human MSC | NA | 3X10^6^ | NA | Yes in mNSS | Brain Res. 2004;1030:19-27. |
| 3h | Wistar rat | Permanent MCAO | PIGF-human MSC and Human MSC | NA | 1X10^7^ | Yes | Yes in limb placement test and the treadmill stress test | Brain. 2006;129:2734-2745. |
| 1h | SD rat | Permanent  MCAO | Human MSC | 5 | 2X10^6^ | Yes | Yes in rotarod test and mNSS | Current study |

MCAO = middle cerebral artery occlusion, MSC = mesenchymal stem cell, SD = Sprague-Dawley, and PIGF = placenta growth factor.
